# Amphetamines, Atomoxetine and the Risk of Serious Cardiovascular Events in Adults

**DOI:** 10.1371/journal.pone.0052991

**Published:** 2013-01-30

**Authors:** Hedi Schelleman, Warren B. Bilker, Stephen E. Kimmel, Gregory W. Daniel, Craig Newcomb, James P. Guevara, Mark J. Cziraky, Brian L. Strom, Sean Hennessy

**Affiliations:** 1 Center for Clinical Epidemiology and Biostatistics, and Department of Biostatistics & Epidemiology, Perelman School of Medicine at the University of Pennsylvania, Philadelphia, Pennsylvania, United States of America; 2 Center for Pharmacoepidemiololgy Research and Training, Perelman School of Medicine at the University of Pennsylvania, Philadelphia, Pennsylvania, United States of America; 3 Department of Medicine, Perelman School of Medicine at the University of Pennsylvania, Philadelphia, Pennsylvania, United States of America; 4 Center for Therapeutic Effectiveness Research, Perelman School of Medicine at the University of Pennsylvania, Philadelphia, Pennsylvania, United States of America; 5 Engelberg Center for Health Care Reform, The Brookings Institution, Washington, District of Columbia, United States of America; 6 PolicyLab: Center to Bridge Research, Practice, and Policy, Children's Hospital of Philadelphia, Philadelphia, Pennsylvania, United States of America; 7 HealthCore, Inc., Wilmington, Delaware, United States of America; University of Manitoba, Canada

## Abstract

**Main Objective:**

To compare the incidence rates of serious cardiovascular events in adult initiators of amphetamines or atomoxetine to rates in non-users.

**Methods:**

This was a retrospective cohort study of new amphetamines (n = 38,586) or atomoxetine (n = 20,995) users. Each medication user was matched to up to four non-users on age, gender, data source, and state (n = 238,183). The following events were primary outcomes of interest 1) sudden death or ventricular arrhythmia, 2) stroke, 3) myocardial infarction, 4) a composite endpoint of stroke or myocardial infarction. Cox proportional hazard regression was used to calculate propensity-adjusted hazard ratios for amphetamines versus matched non-users and atomoxetine versus matched non-users, with intracluster dependence within matched sets accounted for using a robust sandwich estimator.

**Results:**

The propensity-score adjusted hazard ratio for amphetamines use versus non-use was 1.18 (95% CI: 0.55–2.54) for sudden death/ventricular arrhythmia, 0.80 (95% CI: 0.44–1.47) for stroke, 0.75 (95% CI: 0.42–1.35) for myocardial infarction, and 0.78 (95% CI: 0.51–1.19) for stroke/myocardial infarction. The propensity-score adjusted hazard ratio for atomoxetine use versus non-use was 0.41 (95% CI: 0.10–1.75) for sudden death/ventricular arrhythmia, 1.30 (95% CI: 0.52–3.29) for stroke, 0.56 (95% CI: 0.16–2.00) for myocardial infarction, and 0.92 (95% CI: 0.44–1.92) for stroke/myocardial infarction.

**Conclusions:**

Initiation of amphetamines or atomoxetine was not associated with an elevated risk of serious cardiovascular events. However, some of the confidence intervals do not exclude modest elevated risks, e.g. for sudden death/ventricular arrhythmia.

## Introduction

Approximately 1.5 million adults use attention deficit and hyperactivity disorder (ADHD) medications (amphetamines, atomoxetine, and methylphenidate) in the US [1]. These medications have modest effect on blood pressure and heart rate [2], which might increase the risk of cardiovascular events. Post marketing spontaneous reports of myocardial infarction (MI), sudden cardiac death, and stroke while using ADHD medications have led to concern about the cardiovascular safety of these drugs [1].

A recent study found no evidence of an elevated incidence of serious cardiovascular events among adults while using ADHD medications [3]. However, a modest increased risk could not be excluded because of wide 95% confidence intervals. In our prior publication examining methylphenidate, there was a 1.8-fold risk of sudden death or ventricular arrhythmia among adult initiators of methylphenidate, but no increased risk for stroke, MI, or a composite end-point of stroke and MI [4].

The primary aim of this paper is to compare the incidence rates of serious cardiovascular events in adult initiators of amphetamines or atomoxetine to rates in non-users. Our a priori alternative (i.e., non-null) hypothesis was that amphetamines and atomoxetine would be associated with an increased risk of cardiovascular events.

## Methods

This was a cohort study nested within a five-state Medicaid database (1999–2003) and the 14-state HealthCore Integrated Research Database (HIRD 2001–2006). Medicare data were obtained for all Medicaid-Medicare dual eligibles. Prior publications have reported in detail on the study's design and rationale [5], the results in children/adolescents [6], and in adult methylphenidate users [4].

The study was approved by the University of Pennsylvania's Committee on Studies Involving Human Beings, which granted waivers of the requirements for informed consent and Health Insurance Probability and Accountability Act (HIPAA) authorization.

### Study Subjects and Eligible Person-Time

All enrollees 18 years and older who initiated treatment with amphetamines or atomoxetine were identified. Incident user was defined as at least 180 days of observation before the first observed amphetamine or atomoxetine prescription. They were matched on data source, state, sex, and age in six-year age bands up to four non-users who were 18 years and older.

Baseline characteristics (listed in Appendix S1) were ascertained 180-days prior to the start of follow-up. For new amphetamines or atomoxetine users follow-up started when the first amphetamine or atomoxetine prescription was dispensed after an enrollment period of ≥180 days with no ADHD drug exposure. All person-time during an active amphetamine or atomoxetine prescription was included in the analyses. The median duration of a prescription was according to the days' supply field 30 days, and this was comparable to the number of days between fill dates of consecutive prescriptions (32 to 38 days). Therefore, we assumed a prescription lasted 30 days unless a subsequent prescription was dispensed earlier. In sensitivity analyses, to allow for patients non-adherence, we assumed that each prescription lasted a maximum of 60, 90, and 120 days. For non-users follow-up started 180 days after enrollment in the health care plan.

Follow-up ended with the earliest of: 1) the first event of interest; 2) death; 3) disenrollment from the health care plan; 4) the end of the study period; or 5) the end of the last observed amphetamine or atomoxetine prescription or when a person switched to or added a different ADHD medication (only for users). In addition, follow-up ended for HealthCore enrollees when they turned 65 years of age, which is when Medicare eligibility generally begins.

### Outcomes of interest

The following pre-specified incident events were studied as primary outcomes of interest: 1) hospitalization or emergency department visit with a first-listed (principal) diagnosis of sudden cardiac death or ventricular arrhythmia (ICD-9 codes: 427.1, 427.4, 427.41, 427.42, 427.5, 798.1, 798.2); 2) hospitalization with a first-listed diagnosis of stroke (ICD-9 codes: 430, 431, 433.x1, 434 [excluding 434.x0], 436); 3) hospitalization with a first-listed diagnosis of myocardial infarction (MI; ICD-9 code: 410); and 4) a composite of hospitalization with either a first-listed diagnosis of stroke or myocardial infarction. The rationale for considering sudden cardiac death and ventricular arrhythmia as a composite outcome is that sudden cardiac death is often due to undocumented ventricular arrhythmia [7], and considered presumed arrhythmic death. The rationale for combining MI and stroke as a composite outcome is to have more statistical precision to identify an increased cardiovascular risk, which might be due to moderate increase in blood pressure.

The ICD-9 code lists used to identify the events have positive predictive values >70% (85% for sudden death/ventricular arrhythmia [8], 70% for stroke [9]–[13], and >90% for myocardial infarction [14]–[18]). An event with a secondary-listed ICD-9 code for one of these above mentioned events resulted in ending the follow-up time for that outcome, because these outcomes are more likely to represent in-hospital events, which are not of interest in this study of outpatient drug use.

Pre-specified secondary outcomes were all-cause death and non-suicide death (deaths excluding ICD-10 codes X60-X84 and Y87.0). Deaths were ascertained using the Social Security Administration Death Master File and causes of death from the National Death Index.

### Statistical analyses

Age-standardized incidence rates (IRs) with 95% confidence intervals (CIs) were calculated using Stata version 11 (StataCorp LP, College Station, Texas). Cox proportional hazard regression [19] was used to calculate minimally-adjusted hazard ratios (HRs) for amphetamines versus matched non-users and atomoxetine versus matched non-users, with intracluster dependence within matched sets accounted for using a robust sandwich estimator [20], [21]. Because of the low number of events, a post-hoc propensity score analyses was performed [22]. We included all potential confounding factors listed in Appendix S1 and S2 and adjusted by propensity score decile.

In a sensitivity analysis data were limited based on follow-up time (1–180 days) to determine whether differences in follow-up time influenced the results.

Proportional hazard analyses were performed using SAS version 9.2 (SAS Institute, Cary, North Carolina).

## Results

In total 38,586 amphetamine initiators and 20,995 atomoxetine initiators were identified and matched to 238,183 non-users of ADHD medications. Median follow-up was shorter among amphetamine initiators (88 days) and atomoxetine initiators (60 days) than among non-users (non-users matched to amphetamine initiators  = 519 days, non-users matched to atomoxetine users  = 520 days). Table 1 presents the baseline demographics and disease characteristics of the incident users and matched non-users. Initiators of amphetamines and atomoxetine were more likely than non-users to have pre-existing cardiovascular and psychiatric diagnoses.

**Table 1:** Baseline characteristics of incident ADHD medication users and matched non-ADHD medication users, stratified by type of ADHD medication class.

Baseline variables	AMPHETAMINES USERS AND MATCHED NON-USERS				ATOMOXETINE USERS AND MATCHED NON-USERS				
	Amphetamines users N = 38,586		Non-users N = 154,319		Atomoxetine users N = 20,995		Non-users N = 83,964		
	N	(%)	N	(%)	N	(%)		N	(%)
Age in years									
18–47	30,770	(79.7)	123,076	(79.8)	16,505	(78.6)	66,020		(78.6)
48–64	7,493	(19.4)	29,951	(19.4)	4,368	(20.8)	17,456		(20.8)
65+	323	(0.8)	1,292	(0.8)	122	(0.6)	488		(0.6)
Female	20,889	(54.1)	83,543	(54.1)	10,490	(50.0)	41,948		(50.0)
HealthCore Integrated Research Database enrollee	31,853	(82.6)	127,404	(82.6)	16,805	(80.0)	67,216		(80.1)
Race *									
White	4,876	(72.4)	11,608	(43.1)	3,173	(75.7)	8,107		(48.4)
Black	519	(7.7)	5,668	(21.1)	357	(8.5)	3,731		(22.3)
Hispanic	538	(8.0)	5,470	(20.3)	257	(6.1)	2,474		(14.8)
Other	800	(11.9)	4,169	(15.5)	403	(9.6)	2,436		(14.5)
An inpatient or outpatient claim	33,012	(85.6)	103,999	(67.4)	18,878	(89.9)	56,906		(67.8)
Cardiovascular disease/risk factor †	7,071	(18.3)	22,648	(14.7)	4,447	(21.2)	13,362		(15.9)
Treatment for cardiovascular disease/risk factor ‡	6,054	(15.7)	18,344	(11.9)	4,214	(20.1)	11,123		(13.2)
Anxiety	3,495	(9.1)	2,787	(1.8)	2,367	(11.3)	1,601		(1.9)
Asthma	1,560	(4.0)	3,217	(2.1)	1,081	(5.1)	1,911		(2.3)
Bipolar disease	2,041	(5.3)	804	(0.5)	1,670	(8.0)	478		(0.6)
Congenital heart disease	68	(0.2)	221	(0.1)	25	(0.1)	103		(0.1)
COPD	252	(0.7)	713	(0.5)	176	(0.8)	346		(0.4)
Cancer	1,580	(4.1)	3,925	(2.5)	881	(4.2)	2,495		(3.0)
Depression	10,222	(26.5)	6,290	(4.1)	6,339	(30.2)	3,741		(4.5)
Epilepsy	274	(0.7)	505	(0.3)	175	(0.8)	333		(0.4)
HIV	396	(1.0)	720	(0.5)	184	(0.9)	516		(0.6)
Kidney disease	115	(0.3)	465	(0.3)	53	(0.3)	292		(0.3)
Liver disease	834	(2.2)	1,727	(1.1)	565	(2.7)	1,070		(1.3)
Narcolepsy	279	(0.7)	20	(0.0)	18	(0.1)	12		(0.0)
Obesity	1,090	(2.8)	1,751	(1.1)	572	(2.7)	1,014		(1.2)
Psychosis	804	(2.1)	1,284	(0.8)	761	(3.6)	836		(1.0)

* Only available for the Medicaid population.

† Cardiovascular disease/risk factor  =  sudden death/ventricular arrhythmia, myocardial infarction; stroke, cardiomyopathy, diabetes mellitus, heart failure, hypercholesterolemia, hypertension, ischemic heart disease.

‡ Treatment for cardiovascular disease/risk factor  =  use of angiotensin-converting enzyme inhibitor, use of aldosterone inhibitor, use of antiadrenergic agent, use of antiarrhythmic agent, use of antidiabetic agent, use of antihyperlipidemic agent, use of beta-blocker, use of calcium channel blocker, use of loop diuretics, use of nitrate, use of thiazide diuretic, and use of vasodilator.

Table 2 presents the age-standardized incidence rates and minimally-adjusted and propensity score-adjusted HRs for the study outcomes. Of the SD/VA outcomes, 71% had a principal sudden cardiac death diagnosis and 29% had a principal ventricular arrhythmia diagnosis. None of the propensity score-adjusted HRs differed statistically from 1, except for the risk of all-cause death among atomoxetine initiators (HR  = 0.50; 95% CI  = 0.28–0.89). The results from non-suicide deaths were almost identical to those for all-cause death (data not shown). None of the hazard ratios differed statistically between the Medicaid and HealthCore populations. Assuming the maximum duration of a prescription was 60, 90, or 120 days (instead of 30 days) resulted in similar results (Appendix S2). Limiting the follow-up time to 1–180 days produced similar results as those shown in table 1 (data not shown), although the hazard ratio for the inverse association between atomoxetine use and all-cause death was no longer statistically significant (HR  = 0.61; 95% CI: 0.31–2.00).

**Table 2:** Incident ADHD medication users versus non-users and the incidence rates of serious cardiovascular events and all-cause death.

Class of ADHD medication	Number of events in users	Age-standardized incidence rate per 1,000 person-years (95% CI)*	Number of events in non-users	Age-standardized incidence rate per 1,000 person-years (95% CI)†	Minimally-adjusted hazard ratio‡	Propensity Score-adjusted hazard ratio§
**Sudden death/ventricular arrhythmia**						
Amphetamines	**	0.76 (0.34–1.44)	133	0.68 (0.57–0.80)	1.55 (0.77–3.16)	1.18 (0.55–2.54)
Atomoxetine	**	0.46 (0.04–1.70)	103	0.96 (0.78–1.16)	0.54 (0.13–2.29)	0.41 (0.10–1.75)
**Stroke**						
Amphetamines	12	1.33 (0.68–2.33)	250	1.42 (1.25–1.61)	0.94 (0.52–1.70)	0.80 (0.44–1.47)
Atomoxetine	**	1.73 (0.62–3.79)	138	1.45 (1.22–1.71)	1.43 (0.61–3.35)	1.30 (0.52–3.29)
**Myocardial infarction**						
Amphetamines	13	1.31 (0.70–2.25)	309	1.68 (1.50–1.88)	0.88 (0.50–1.56)	0.75 (0.42–1.35)
Atomoxetine	**	0.81 (0.15–2.39)	166	1.66 (1.41–1.93)	0.54 (0.17–1.72)	0.56 (0.16–2.00)
**Composite outcome of stroke and myocardial infarction**						
Amphetamines	25	2.62 (1.69–3.87)	547	3.03 (2.78–3.29)	0.93 (0.62–1.40)	0.78 (0.51–1.19)
Atomoxetine	**	2.47 (1.12–4.71)	293	2.99 (2.65–3.35)	0.93 (0.47–1.84)	0.92 (0.44–1.92)
**All-cause death**						
Amphetamines	69	9.05 (7.04–11.46)	1,011	8.20 (7.70–8.72)	1.41 (1.09–1.82)	0.92 (0.71–1.19)
Atomoxetine	13	4.95 (2.63–8.49)	578	9.18 (8.44–9.95)	0.77 (0.44–1.37)	0.50 (0.28–0.89)

* Standardized based on the major age distribution (18–47, 48–64, and 65+) of the overall incident user group.

† Standardized based on the major age distribution (18–47, 48–64, and 65+) of the overall non-user group who were matched to incident users.

‡ Adjusted for continuous age and data source.

§ In the propensity score were all variables included that were listed in Appendix S1.

** Count omitted per Centers for Medicare & Medicaid Services Privacy Guideline policy.

## Discussion

In this study, initiation of amphetamines or atomoxetine among adults was not associated with an increased risk of serious cardiovascular events compared to non-ADHD medication users. Our results are consistent with another cohort study using data from administrative claims [3]. Further, Holick et al. [23] found a higher risk of transient ischemic attack (HR = 3.44; 95% CI = 1.13–10.60), but not of cerebrovascular accident (HR  = 0.71; 95% CI  = 0.34–1.47), in adult new users of stimulants. Our results, differ from our previously reported association between initiation of methylphenidate and a nearly doubling of the rate of sudden death or ventricular arrhythmia among adults [4]. We are unaware of any distinctive pharmacologic characteristic of methylphenidate compared to amphetamines and atomoxetine that would lead to a unique risk for this therapeutic class. The absence of an apparent dose-response relationship between methylphenidate and sudden death/ventricular arrhythmia in our prior publication does not support a causal relationship [4].

This study has several limitations. Important is the limited number of events that were identified. Therefore, the study did not have enough precision to allow stratified analyses (e.g., examination of dose). In addition, despite the large size of the database, modest elevated risks cannot be ruled out for all of the evaluated associations. Nonetheless, it is reassuring that in two large studies (Habel and colleagues [3] and our study) no association was found between initiation of amphetamines or atomoxetine and serious cardiovascular events.

A second important limitation is that this study was non-randomized, which leaves the potential for unmeasured confounding. Administrative data do not record all factors that one would like to examine as potential confounders, e.g., smoking, blood pressure, nonprescription aspirin use, substance misuse, physical activity. The numeric imbalances in favor of the exposed group seen in this and other studies suggest that persons treated with ADHD medications may have had a lower baseline risk of events than the control groups. Measured baseline factors were more imbalanced for methylphenidate vs. non-users than for amphetamines and atomoxetine vs. non-users. It is possible that unmeasured confounding factors could have had the same pattern.

A third limitation is that this study relied on the accuracy of claims diagnosis. The diagnosis codes used, however, had high positive predictive values in adults (>70%). Nonetheless, our results may have been biased towards the null because of misclassification of outcomes. In addition, because many strokes are coded with diagnoses that do not distinguish between hemorrhagic and ischemic mechanisms, we were unable to distinguish between these mechanisms. By combining the two mechanisms, the results might have been biased towards the null, because drugs that raise blood pressure are more likely to increase the risk of hemorrhagic stroke. However, there were too few hemorrhagic strokes to analyze this sub-outcome.

A fourth limitation is that exposure to amphetamines and atomoxetine was assessed based on the fill date of a prescription, because actual data on whether a dose was taken is not available in claims databases. Thus, we cannot account for non-adherence or drug holidays. In the primary analyses we assumed that each prescription lasted a maximum of 30 days. To reduce the potential for missing an increased cardiovascular risk, if e.g. any cardiovascular effects of ADHD medications was persist, sensitivity analyses were performed elongating the maximum duration of a prescription (60, 90 and 120 days).

In summary, in this non-randomized cohort study of Medicaid and commercial insurance enrollees, initiation of amphetamines or atomoxetine was not associated with an elevated risk of serious cardiovascular events, but some of the confidence intervals do not exclude modest elevated risks, e.g. for sudden death/ventricular arrhythmia.

## Supporting Information

Appendix S1 **Variables examined as potential confounders.**
(DOCX)

Appendix S2 **Incident ADHD medication users versus non-users and the incidence rates of serious cardiovascular events and all-cause death; different criteria for the maximum duration of a prescription.**
(DOCX)
