# Supplementary material for: Amphetamines, Atomoxetine and the Risk of Serious Cardiovascular Events in Adults
Source: PLoS One. 2013 Jan 30;8(1):e52991. doi: 10.1371/journal.pone.0052991 (PMC3559703; doi:10.1371/journal.pone.0052991)
Supplement: Appendix S1 — Variables examined as potential confounders. (DOCX) [file pone.0052991.s001.docx]

Appendix S1. Variables examined as potential confounders

| **Demographic variables** | **Diagnosis (continued)** | **Drugs** |
| --- | --- | --- |
| Age | Encopresis | ACE inhibitor |
| Gender | Enuresis | Aldosterone inhibitor |
| Race* | Epilepsy | Alpha-1 blocker |
| State | Infantile cerebral palsy | Anorexient agent |
| Data source | HIV | Antiadrenergic agent |
| Calendar year | Heart failure | Antiarrhythmic agent |
| Nursing home residence* | Hypercholesterolemia | Antidiabetic agent |
| An inpatient or outpatient claim | Hypertension | Antihyperlipidemic agent |
| **Diagnosis** | Hypothyroidism | Antipsychotic dose |
| Acquired or hereditary anemia | Ischemic heart disease | Antiseizure agent |
| Adjustment disorder | Kidney disease | Anxiolytic agent |
| Alcohol use/abuse | Learning disorder | Aspirin |
| Anxiety | Liver disease | Beta-blocker |
| Arrhythmia | Marfan syndrome | Bronchodilator |
| Asthma | Myocardial infarction | Bupropion |
| Autism | Muscular dystrophy | COX-2 inhibitors |
| Bipolar disease | Narcolepsy | Calcium channel blocker |
| COPD | Obesity | Immunosuppressive agent |
| Bronchitis | Obsessive compulsive disorder | Inhaled corticosteroid |
| Cancer | Oppositional defiant disorder | Inotropic agent |
| Capillaries disease | Osteoarthritis | Loop diuretic |
| Cardiomyopathy | Osteogenesis imperfecta | NSAID |
| Cerebral degeneration | Other congenital malformations | Nitrate |
| Chromosomal abnormalities | Other hereditary immune diseases | Oral corticosteroid |
| Conduct disorder | Other metabolic disorders | SSRI/SSNRI |
| Coagulation defects | Psychosis | Thiazide diuretic |
| Congenital heart disease | Rheumatoid arthritis | Thyroid |
| Cystic fibrosis | SD/VA | Tricyclic antidepressant |
| Depression / depressive disorder | Smoking | Vasodilators |
| Diabetes mellitus | Stroke | Warfarin |
| Electrocardiogram | Tic disorder | Xanthine derivate |
| Emphysema | Valvular heart disease |  |

ACE = angiotensin-converting enzyme; COPD = chronic obstructive pulmonary disease; COX = cyclooxygenase; HIV = human immunodeficiency virus; NSAID = non-steroidal anti-inflammatory agent; SD/VA = sudden death/ventricular arrhythmia; SS(N)RI = selective serotonin(-norepinephrine) reuptake inhibitor

* Only available in Medicaid/Medicare database
