# Supplementary material for: Amphetamines, Atomoxetine and the Risk of Serious Cardiovascular Events in Adults
Source: PLoS One. 2013 Jan 30;8(1):e52991. doi: 10.1371/journal.pone.0052991 (PMC3559703; doi:10.1371/journal.pone.0052991)
Supplement: Appendix S2 — Incident ADHD medication users versus non-users and the incidence rates of serious cardiovascular events and all-cause death; different criteria for the maximum duration of a prescription. (DOCX) [file pone.0052991.s002.docx]

Appendix S2. Incident ADHD medication users versus non-users and the incidence rates of serious cardiovascular events and all-cause death; different criteria for the maximum duration of a prescription.

| Class of ADHD medication | **Prescription lasted max. 30 days (main analysis)**  Propensity Score-adjusted hazard ratio* | **Prescription lasted max. 60 days**  Propensity Score-adjusted hazard ratio* | **Prescription lasted max. 90 days**  Propensity Score-adjusted hazard ratio* | **Prescription lasted max. 120 days**  Propensity Score-adjusted hazard ratio* |
| --- | --- | --- | --- | --- |
| **Sudden death / ventricular arrhythmia** | | | | |
| Amphetamines | 1.18 (0.55-2.54) | 0.99 (0.50-1.97) | 1.00 (0.54-1.86) | 1.05 (0.59-1.90) |
| Atomoxetine | 0.41 (0.10-1.75) | 0.30 (0.07-1.27) | 0.39 (0.12-1.23) | 0.47 (0.17-1.27) |
| **Stroke** | | | | |
| Amphetamines | 0.80 (0.44-1.47) | 0.67 (0.38-1.19) | 0.77 (0.48-1.25) | 0.81 (0.52-1.26) |
| Atomoxetine | 1.30 (0.52-3.29) | 1.04 (0.43-2.50) | 1.27 (0.60-2.71) | 1.15 (0.54-2.42) |
| **Myocardial infarction** | | | | |
| Amphetamines | 0.75 (0.42-1.35) | 0.64 (0.38-1.08) | 0.68 (0.42-1.12) | 0.77 (0.48-1.21) |
| Atomoxetine | 0.56 (0.16-2.00) | 0.54 (0.18-1.62) | 0.56 (0.21-1.49) | 0.50 (0.19-1.33) |
| **Composite outcome of stroke and myocardial infarction** | | | | |
| Amphetamines | 0.78 (0.51-1.19) | 0.66 (0.45-0.98) | 0.73 (0.52-1.04) | 0.79 (0.58-1.10) |
| Atomoxetine | 0.92 (0.44-1.92) | 0.78 (0.40-1.54) | 0.90 (0.50-1.62) | 0.81 (0.45-1.45) |
| **All-cause death** | | | | |
| Amphetamines | 0.92 (0.71-1.19) | 0.94 (0.76-1.18) | 1.04 (0.85-1.28) | 1.07 (0.88-1.30) |
| Atomoxetine | 0.50 (0.28-0.89) | 0.52 (0.32-0.85) | 0.64 (0.42-0.98) | 0.64 (0.43-0.96) |

* In the propensity score were all variables included that were listed in Appendix S1
